# Legal Notes for Institutional Workers

**Published:** 1911-11-18

**Authors:** 


### LEGAL NOTES FOR INSTITUTIONAL WORKERS.

(The Editor will be pleased to answer Legal queries in this column.)

### HOSPITALS AND WATER-SUPPLY.

Those who have to pay the institutional water-rate are aware of the fact that there are two methods of charging. The water company may demand an annual payment which varies practically with the rateable value, or they may charge by measure. As a general rule charge by measure is insisted on, unless the premises served are a "private dwelling-house," or unless the water is used for "domestic purposes." Is a hospital a "private dwelling-house"? Does it use "water for domestic purposes"? In a recent case Mr. Justice Eve has held that a workhouse is not a private dwelling-house. What, then, would be the position of a hospital? Mr. Justice Eve said: "It seems to me that in considering the words 'private dwelling-house' you cannot say that the word 'private' does not differentiate dwelling-houses. If that were not so, the section would extend not only to private dwelling-houses but to business premises and public institutions." A public hospital, *e.g.* St. Bartholomew's, would probably be held not to be a private dwelling-house, while a nursing home would come within the definition. The phrase "domestic purposes" has been frequently discussed in courts of law. That "domestic purposes" is a phrase of wide meaning is clear from a case in which it was held that a doctor who paid a fixed charge might use water for washing his motor. The use of water in a hospital has not yet been discussed in this connection.

### A CHARGE OF MALINGERING.

Employers who are met with claims for compensation are sometimes driven to protecting themselves by alleging that the claimant is either a malingerer or the victim of self-inflicted injuries. A charge of malingering must not be lightly made; and even when the facts of a case are difficult to explain in any other way it is by no means easy to persuade a judge that the claimant has been guilty of this curious offence. In a recent case at Manchester a hospital nurse sought compensation. Although malingering was not actually suggested by the employers, it was part of their case that the applicant had occasioned the injury by trying a treatment of her own. On July 12, 1910, she scalded her wrist. She was treated in the Liverpool Infirmary. Her full wages were paid until May 1911. It was proved that she was suffering from lymphædema, which, according to the evidence of a doctor called on her behalf, could be caused by a scald. A doctor called on behalf of the employers (the Guardians of the South Manchester Union) said that, in his view, some physical condition must have caused the obstruction of the vessels of the wrist, while the assistant medical officer at the Withington Workhouse, where the applicant was employed, declared that when he saw her she was doing something to cause it. It had been suggested, in cross-examination, that she had tightly bandaged her hand. This she denied. The visiting surgeon at the hospital was of the same opinion. He told the judge that such conduct might be caused by hysteria. The judge observed that this amounted to saying that she had been causing herself pain for eighteen months, and he refused to believe it. In the event, he made an award of half wages.

### THE HOSPITAL STAFF AND WORKMEN'S COMPENSATION.

It is now fairly well established that members of the staff of a voluntary hospital are workmen within the meaning of the Compensation Act. But for what accidents to them is the institution responsible? The question is important, for people do not pay compensation or even insurance premiums for fun. To take out a policy in respect of the entire staff is a serious matter for the voluntary hospital. One must consider the legal effect of the words "arising out of and in the course of." If an operating surgeon were to cut his finger, and sepsis were to supervene, that would clearly be an accident arising out of and in the course of his employment. The $x$-ray case, which was recently noticed in this journal, is at the other extreme, the house surgeon being a mere volunteer in what he did. It is possible to think of many intermediate cases. Suppose, for instance, that it was necessary to apply a sinapism to a patient's back, and a new batch of plasters came in of which the efficacy was unknown. The house physician puts one on his own arm and is injured. Did the accident arise out of and in the course of his employment? Here is a question for a bench of judges! It would seem that each case must be decided on its own peculiar facts; but to avoid litigation every member of the junior staff should be insured under the Act.

### LEGAL AID SOCIETIES.

The question must often arise in the out-patient department—Is there no person or society which could take up the case of this unfortunate man, and assist him to assert his legal rights? It is not generally known that in many populous centres there is a poor man's lawyer, either a barrister or a solicitor, who is ready to give advice gratis. At Mansfield House, for instance, assistance of this kind is given to those who live in the East End. Those who minister to the poor in the slums of London have found that a judicious letter written or drafted by a legal friend often brings about a favourable settlement of a claim. But there are grave practical objections to the lawyer doing more than advise and write a letter, unless the usual relationship of solicitor and client exists. Who is to pay the out-of-pocket expenses which must be incurred? Again, no counsel at the Bar can go into Court on the "No cure, no pay principle." In the matter of legal aid, Scotland is a long way ahead of England, for beyond the Tweed special rules are in force to enable members of either branch of the profession to help the impecunious litigant.

